# Mechanistic Understanding of the Palmitoylation of G_o_ Protein in the Allosteric Regulation of Adhesion Receptor GPR97

**DOI:** 10.3390/pharmaceutics14091856

**Published:** 2022-09-02

**Authors:** Hao Zhang, Guojun Chu, Gaoming Wang, Min Yao, Shaoyong Lu, Ting Chen

**Affiliations:** 1 Department of Plastic and Reconstructive Surgery, Shanghai Ninth People’s Hospital, Shanghai Jiao Tong University, School of Medicine, Shanghai 200011, China; 2 Department of Cardiology, Changzheng Hospital, Naval Medical University, Shanghai 200003, China; 3 Medicinal Chemistry and Bioinformatics Center, Shanghai Jiao Tong University, School of Medicine, Shanghai 200025, China

**Keywords:** G-protein-coupled receptor (GPCR), GPR97, palmitoylation, molecular dynamics simulation, Markov state model

## Abstract

Adhesion G-protein-coupled receptors (aGPCRs)—a major family of GPCRs—play critical roles in the regulation of tissue development and cancer progression. The orphan receptor GPR97, activated by glucocorticoid stress hormones, is a prototypical aGPCR. Although it has been established that the palmitoylation of the C-terminal G_o_ protein is essential for G_o_’s efficient engagement with the active GPR97, the detailed allosteric mechanism remains to be clarified. Hence, we performed extensive large-scale molecular dynamics (MD) simulations of the GPR97−G_o_ complex in the presence or absence of G_o_ palmitoylation. The conformational landscapes analyzed by Markov state models revealed that the overall conformation of GPR97 is preferred to be fully active when interacting with palmitoylated G_o_ protein. Structural and energetic analyses indicated that the palmitoylation of G_o_ can allosterically stabilize the critical residues in the ligand-binding pocket of GPR97 and increase the affinity of the ligand for GPR97. Furthermore, the community network analysis suggests that the palmitoylation of G_o_ not only allosterically strengthens the internal interactions between G_αo_ and G_βγ_, but also enhances the coupling between G_o_ and GPR97. Our study provides mechanistic insights into the regulation of aGPCRs via post-translational modifications of the G_o_ protein, and offers guidance for future drug design of aGPCRs.

## 1. Introduction

G-protein-coupled receptors (GPCRs) constitute the largest family of cell-surface signaling receptors in mammalian cells, regulating numerous cellular and physiological processes [1]. GPCRs represent the largest class of drug targets, as their dysregulated signaling has been associated with a broad spectrum of human diseases, including central nervous system disorders; cardiac, metabolic, and inflammatory diseases; and cancers [2]. Human adhesion GPCRs (aGPCRs)—a major family of GPCRs—contain 33 members; aGPCRs are known for a large ectodomain containing the GAIN domain [3]. When aGPCRs are activated by agonists, the GAIN domain functions mutually with the seven-transmembrane (7TM) bundle [4,5]. Then, aGPCRs couple to the heterotrimeric G proteins such as the G_o_ protein at the plasma membrane, and activate downstream signaling [6,7]. The aGPCRs are key molecular switches, regulating diverse physiological responses including brain development, ion–water homeostasis, inflammation, and cell fate determination [8,9]. Mutations in aGPCRs are implicated in numerous human diseases, including vibratory urticaria, bilateral frontoparietal polymicrogyria, chondrogenesis, Usher syndrome, and male infertility [10,11,12]. However, the structural basis of aGPCRs’ activation and their coupling with G proteins remains unclear [13].

The orphan receptor GPR97, encoded by *Adgrg3*, is a member of the aGPCR family [1]. GPR97 is implicated in the progression of experimental autoimmune encephalomyelitis, the fate determination of B lymphocytes, and the development of acute kidney injury [14,15,16]. The 7TM bundle of GPR97 comprises a large crevice that accommodates the G_o_ trimer at the cytoplasmic surface [17]. Glucocorticoid stress hormones such as the anti-inflammatory drug beclomethasone (BCM) and cortisol can activate GPR97 by binding to a pocket within the 7TM domain [1]. Two recent cryo-electron microscopy (cryo-EM) structures of BCM- or cortisol-bound GPR97−G_o_ complexes indicate that there is a palmitoylation at the C351 of the α5 helix at the C-terminus of the G_o_ protein, inserting deeply into the 7TM core of GPR97 [18]. The palmitoylation at the C-terminus is critical for efficient engagement of the G_o_ protein with GPR97 in the active state, but has not been reported previously in other solved GPCR−G protein complex structures [18].

Palmitoylation is a dynamic and reversible post-translational modification (PTM) that is prevalent in GPCRs and their cognate G proteins [19], and is considered to have important regulatory functions [20]. While previous studies largely focused on the palmitoylation of GPCRs [21], the exploration of the palmitoylation of G proteins achieved limited knowledge that the palmitoylation of G proteins contributes to recruitment of the Gα protein to membranes [19,22,23]. Despite the recent first report of the palmitoylation of G proteins in the allosteric regulation of GPCR functions, the role of palmitoylation at the C-terminus of the G_o_ protein in its specific allosteric coupling to GPR97 remains to be clarified.

To characterize the detailed conformational dynamics of GPR97 in different states, we performed extensive large-scale molecular dynamics (MD) simulations of BCM- or cortisol-bound GPR97 in the presence or absence of G_o_ or palmitoylated G_o_. The simulations revealed that the dynamic conformation of the active GPR97 is more stable upon G_o_ binding—especially with palmitoylated G_o_. Dissection of conformational landscapes through Markov state model (MSM) analysis showed that the interaction of G_o_ with GPR97 contributes to the high basal activity of GRP97, while palmitoylation of G_o_ further increases the proportion of active GPR97 conformation. Analysis of key conformational substates indicated that palmitoylation of G_o_ allosterically enhances the interaction between agonist ligands and GPR97. Hence, our results reveal an in-depth mechanistic mechanism underlying the palmitoylation of G_o_ in its specific coupling to GPR97. Although advances in the GPCR structures and pharmacology have improved drug discovery [24], the regulation of GPCR functions by diverse PTMs of G proteins has still received little attention. To the best of our knowledge, this study provides the first dynamic structural insights into GPCR regulation via PTMs of G proteins, and offers guidance for the innovative improvement and refinement of GPCR modulators [25].

## 2. Materials and Methods

### 2.1. Construction of Stimulation Systems

Six systems were constructed: BCM–GPR97, BCM–GPR97–G_o_, BCM–GPR97–palmitoylated G_o_, cortisol–GPR97, cortisol–GPR97–G_o_, and cortisol–GPR97–palmitoylated G_o_. The initial structures for BCM–GPR97–palmitoylated G_o_ (PDB ID: 7D76) and cortisol–GPR97–palmitoylated G_o_ (PDB ID: 7D77) were derived from the Protein Data Bank [18]. We modeled the missing residues in the original crystal profile with available X-ray structures of relevant homologs using the Discover Studio program. According to the BCM–GPR97–palmitoylated G_o_ complex, the structures of BCM–GPR97 and BCM–GPR97–G_o_ were extracted from the complex. Similarly, both the cortisol–GPR97 and Cortisol–GPR97–G_o_ complexes were extracted from the cortisol–GPR97–palmitoylated G_o_ complex.

**Figure 1:**
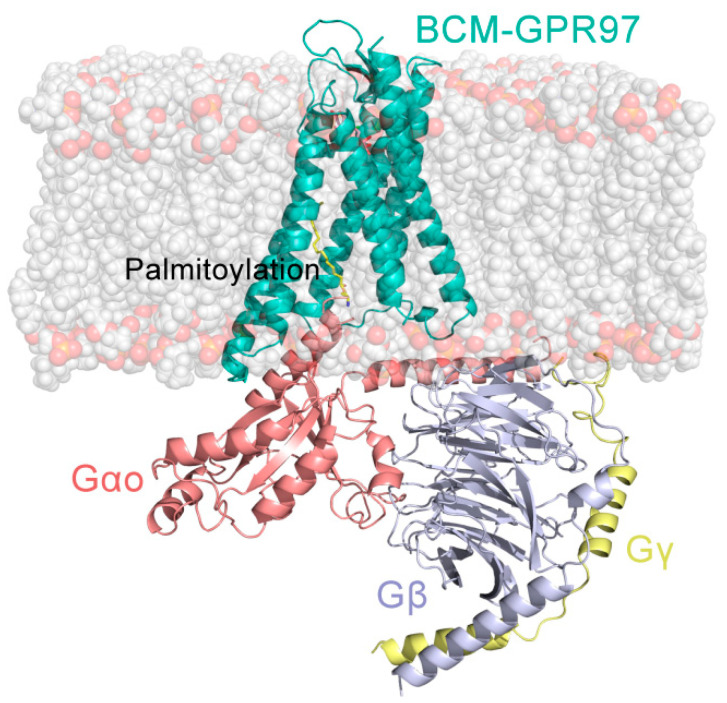
Orthogonal view of the model of the BCM–GPR97–Go complex. GPR97 is shown in light sea green, G_αo_ in salmon, G_β_ in light blue, G_γ_ in yellow, and BCM in pink.

The obtained complexes were first oriented in the Orientations of Proteins in Membranes (OPM) server [26]. These structures were fixed into the DOPC membrane on the CHARMM-GUI server (Figure 1) [27]. Secondly, the systems were implanted in water molecules, with 80 water molecules per lipid molecule. A salt concentration of 0.15 mol/L NaCl was used to balance the charge. Finally, we obtained the coordinates and topologies for AMBER according to the input generation of CHARMM-GUI.

### 2.2. MD Simulations Settings

MD simulations for the six systems were performed with AMBER 18. The initial parameter files for minimizations and simulations were prepared using the AMBER ff14SB force field and general AMBER force field (GAFF) [28,29]. A transferable intermolecular potential three-point (TIP3P) truncated octahedral water box (10 Å) was used for solvation, followed by the addition of counterions for neutralization [30]. Subsequently, 0.15 mol L^−1^ NaCl was added to each system to achieve the required physiological conditions for the proteins.

Next, the six systems were subjected to two-round energy minimizations with the steepest descent and conjugate gradient algorithms. Every system was heated from 0 to 310 K in 300 ps in a canonical ensemble (NVT), with equilibrium runs of 700 ps. Finally, 3 independent MD runs with random initial velocities and a duration of 1 μs were carried out for the six systems. We obtained 18 independent trajectories with a cumulative 18 μs in length. The particle mesh Ewald method was employed for the incorporation of the long-range electrostatic interactions within the systems, and a 10 Å cutoff was introduced for the short-range electrostatics and van der Waals interactions [31]. All covalent bonds engaging hydrogen atoms were restricted using the SHAKE method [32].

### 2.3. Dynamic Cross-Correlation Matrix (DCCM) Analysis

To determine the inter-residue correlations in each system, the DCCM of all protein Cα atoms, which represents the fluctuations in Cα atom coordinates, was calculated with the CPPTRAJ plugin [33] using Equation (1):

(1) $$C_{\mathrm{ij}}=\frac{{\Delta r}_{i}\times {\Delta r}_{j}}{\sqrt{{\left(\Delta r_{i}\right)}^{2}\times {\left({\Delta r}_{j}\right)}^{2}}}$$

where Δr_i_ and Δr_j_ represent the atomic displacement vectors for the ith and jth Cα atoms, respectively.

### 2.4. Principal Component Analysis (PCA)

During MD simulations, PCA is a normal tool used to analyze large-scale collective motions of biological macromolecules. This statistical technique can capture large-amplitude motions of the system by reducing the number of degrees of freedom to a vital subspace set. To identify the system motions, we calculated and diagonalized the covariance matrix of the receptor Cα atoms using the CPPTRAJ plugin of AMBER. Then, a new set of coordinates was generated (named eigenvectors), which were also named principal components (PCs). The eigenvalue is related to the mean square fluctuation contained in the trajectory projected along the eigenvector.

Since the first PC (i.e., PC1) corresponds to the largest-amplitude motion of the system, the dynamics along PC1 are usually considered to be the “essential dynamics” of the system [34]. In this work, the covariance matrix of the protein’s Cα atoms was mass-weighted to compute the protein’s principal motions. Specifically, we projected the sampled conformations corresponding to the trajectories onto the collective coordinate space defined by PC1, in terms of the initial structure of the receptor for each system. Finally, using a plugin in Visual Molecular Dynamics (VMD), the visualization of major motions for every system was presented as porcupine plots.

### 2.5. Markov State Model Construction

Based on the coordinates of GPR97 in the six systems, the PCA of the overall protein backbone throughout the simulation of all systems was calculated and then taken as the input for MSM analysis. The Python library PyEMMA (http://www.emma-project.org/latest/) (accessed on 15 January 2022) was utilized to construct and validate Markov state models (MSMs) with MD simulation data [35]. Based on implied timescale verification, we confirmed that the six systems were Markovian and reliable, with a lag time of 60 ns for 100 microstate models, and a maximum of 100 k-means iterations [36]. Then, the microstates were clustered into three metastates in each system using the PCCA+ algorithm, which was confirmed by the Chapman–Kolmogorov test [37].

Utilizing TPT, we successfully identified the transition probability matrix of the MSMs, and measured the average first-pass time [38]. The structures near the microstate cluster centers of each macrostate were extracted into the trajectories for the corresponding metastates using the MDTraj package [39]. Finally, the representative conformation of each metastate was selected according to the similarity score S_ij_.

(2) $$S_{\mathrm{ij}}=e^{-d_{\mathrm{ij}}/d_{\mathrm{scale}}}$$

In Equation (2), the structure with the highest S_ij_ among the trajectories is the most representative conformation of the metastate. The d_ij_ represents the RMSD between the conformations i and j, while d_scale_ is the standard deviation of d.

### 2.6. Community Network Analysis

Using the NetworkView plugin in VMD [40], we calculated the community organizations among different systems based on the correlation coefficient matrix C_ij_. The whole GPR97 in every system was considered as a group of nodes (assigned to the Cα atom of each residue) connected by edges, which were drawn between nodes that remained within a cutoff distance of 4.5 Å for at least 75% of the simulation process [41,42]. The edge connections between certain nodes were calculated using Equation (3):

d_i,j _ =  −log(|C_i,j_|) (3)

where i and j represent two nodes, and C_ij_ was calculated by Equation (1).

Next, optimal pathways between all pairs of nodes were computed using the Floyd–Warshall algorithm. The gncommunities program was used to determine the substructures of the communities, which embedded the Girvan–Newman divisive algorithm and applied edge betweenness, defined as the number of paired optimal paths. To determine the optimal substructure of the network, the edges with the highest betweenness were iteratively removed from the network, and the remaining edges were recomputed until each node represented an isolated community. Communities with less than three residues were discarded. Connectivity between communities was quantified by the betweenness value.

## 3. Results

We performed 1 μs × 3 independent MD simulations with random initial velocities for BCM–GPR97, BCM–GPR97–G_o_, BCM–GPR97–palmitoylated G_o_, cortisol–GPR97, cortisol–GPR97–G_o_, and cortisol–GPR97–palmitoylated G_o_ systems, leading to a cumulative simulation timescale of 18 μs.

### 3.1. Palmitoylated G_o_ Enhances the Stability of GPR97’s Conformational Dynamics

**Figure 2:**
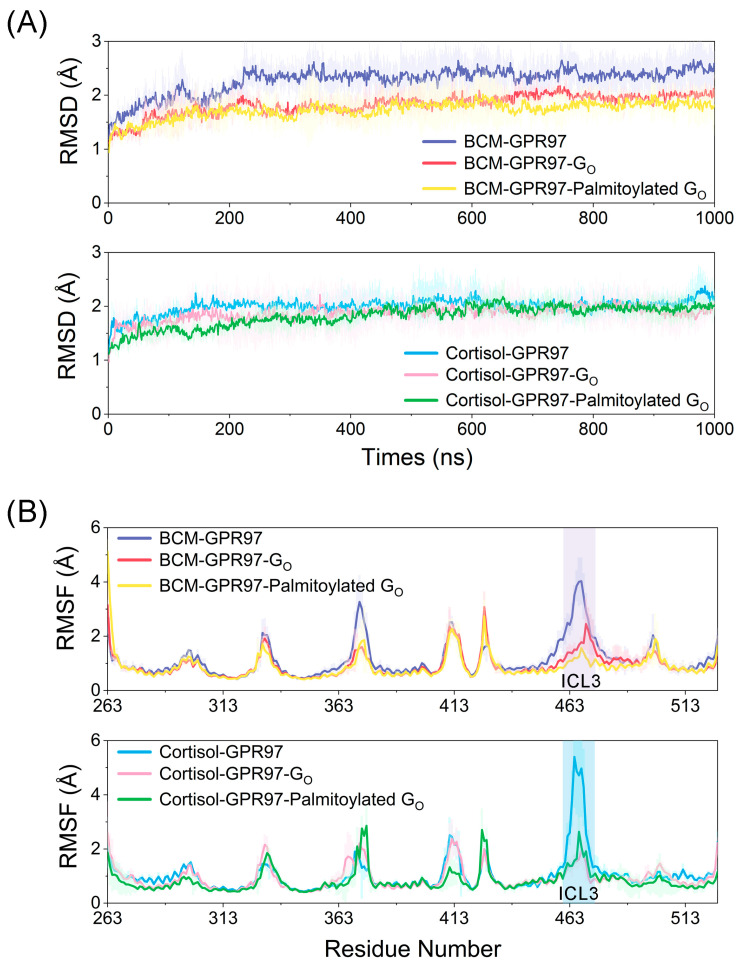
(**A**) The Cα RMSD of GPR97 in the BCM–GPR97 (purple), BCM–GPR97–G_o_ (red), BCM–GPR97–palmitoylated G_o_ (yellow), cortisol–GPR97 (blue), cortisol–GPR97–G_o_ (pink), and cortisol–GPR97–palmitoylated G_o_ (green) systems in 1000 ns MD simulations. (**B**) The Cα RMSF of GPR97 in BCM–GPR97 (purple), BCM–GPR97–G_o_ (red), BCM–GPR97–palmitoylated G_o_ (yellow), cortisol–GPR97 (blue), cortisol–GPR97–G_o_ (pink), and cortisol–GPR97–palmitoylated G_o_ (green) systems in 1000 ns MD simulations (residues 464–472 are ICL3).

The root-mean-square deviation (RMSD) of the Cα atoms of GPR97 in six systems was measured to quantify the dynamic conformational changes throughout the simulations (Figure 2A). The RMSD plots indicated that all six systems reached equilibrium at ~200 ns simulations. The RMSD values of GPR97 in the BCM−GPR97, BCM–GPR97–G_o_ and BCM–GPR97–palmitoylated G_o_ systems were 2.28 ± 0.35 Å, 1.83 ± 0.24 Å, and 1.73 ± 0.28 Å, respectively. Similarly, the RMSD values for the cortisol–GPR97, cortisol–GPR97–G_o_, and cortisol–GPR97–palmitoylated G_o_ systems were 2.00 ± 0.21 Å, 1.86 ± 0.32 Å, and 1.82 ± 0.24 Å, respectively. It should be noted that the RMSD value of GPR97 decreased when complexed with G_o_, with the lowest RMSD values in complex with the palmitoylated G_o_. This indicates that GPR97 in the BCM-/cortisol-bound GPR97–palmitoylated G_o_ states exhibited the most stable conformational dynamics, further supporting the notion that G_o_ binding can stabilize GPR97—especially when the aliphatic chain of palmitoylation is inserted into the 7TM bundle of GPR97.

To investigate the local conformational dynamics of GPR97, the atomic root-mean-square fluctuations (RMSFs) of Cα atoms around their original positions were quantified for each residue (Figure 2B). The RMSF values of GPR97 in the six systems were 1.12 ± 0.69 Å (BCM–GPR97), 0.93 ± 0.48 Å (BCM–GPR97–G_o_), 0.89 ± 0.51 Å (BCM–GPR97–palmitoylated G_o_), 1.04 ± 0.58 Å (cortisol–GPR97), 0.92 ± 0.47 Å (cortisol–GPR97–G_o_), and 0.83 ± 0.46 Å (cortisol–GPR97–palmitoylated G_o_). Typically, GPR97 in the BCM–GPR97–palmitoylated G_o_ and cortisol–GPR97–palmitoylated G_o_ systems displayed a lower RMSF, suggesting that the conformational dynamics of GPR97 were relatively more stable in the presence of palmitoylated G_o_. Notably, intracellular loop 1 (ICL1), which interacts directly with the α4, α5, and β6 of G_o_, displayed relatively higher RMSFs in the BCM–GPR97 and cortisol–GPR97 systems, but decreased when in complex with G_o_—especially the palmitoylated G_o_. This was due to the fact that ICL1 participates in the interaction between GPR97 and G_o_, while the palmitoylation chain at the C351 of the G_o_ α5 helix contributes to the coupling of GPR97 with G_o_.

**Figure 3:**
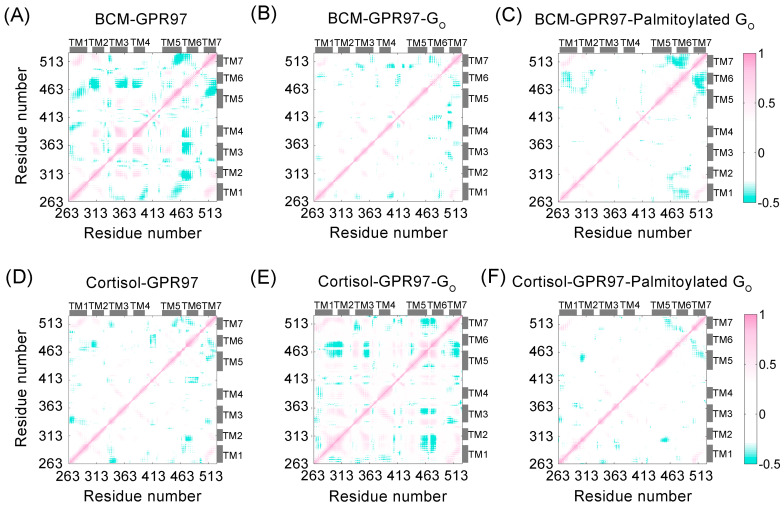
DCCM plots of BCM–GPR97 (**A**), BCM–GPR97–G_o_ (**B**), BCM–GPR97–palmitoylated G_o_ (**C**), cortisol–GPR97 (**D**), cortisol–GPR97–G_o_ (**E**), and cortisol–GPR97–palmitoylated G_o_ (**F**) systems. Positive areas (pink) indicate correlated motion, whereas the negative areas (green) denote anti-correlated motion. The correlation motions with absolute values less than 0.3 are ignored and shown in white.

To explore the intrachain correlations within GPR97 in each system, we calculated residue interactions via dynamic cross-correlation matrices (DCCMs) (Figure 3). Globally, GPR97 in both BCM–GPR97–palmitoylated G_o_ and cortisol–GPR97–palmitoylated G_o_ systems displayed lower values of DCCM. The markedly reduced dynamic correlated motions of GPR97 in the palmitoylated G_o_ systems indicated that palmitoylation of G_o_ might limit the residue motions within GPR97, and the flexibility of GPR97 was reduced, which was consistent with the RMSD and RMSF analyses. In particular, the correlated motions between the TM7 and TM5/TM6 regions were relatively reduced upon G_o_ binding. This may be related to the interruption of kinks between TM5/TM6 and TM7, which are required for GPCR to achieve an active state [43].

### 3.2. The Palmitoylation of G_o_ Contributes to the High Activity of GRP97

**Figure 4:**
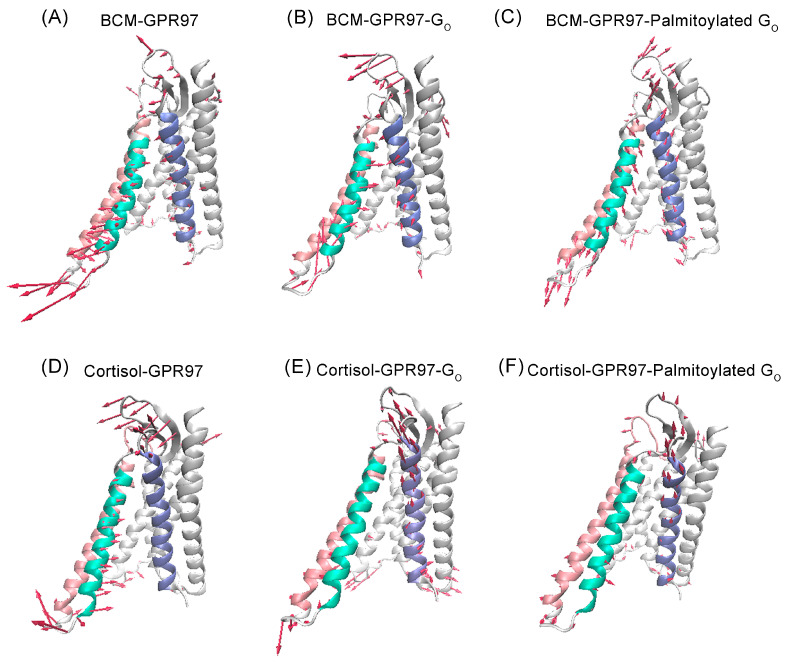
Comparison of the principal modes of motion along PC1 in BCM–GPR97 (**A**), BCM–GPR97–G_o_ (**B**), BCM–GPR97–palmitoylated G_o_ (**C**), cortisol–GPR97 (**D**), cortisol–GPR97–G_o_ (**E**), and cortisol–GPR97–palmitoylated G_o_ (**F**) systems. Red arrows depict the directions of protein motions, while the length of the arrows represents the magnitude of the movements. The TM5, TM6, and TM7 regions are colored red, green, and purple, respectively.

Based on MD trajectories, principal component analysis (PCA) of the overall protein backbone was carried out to characterize the predominant overall conformational variations of GPR97. Porcupine diagrams were constructed, where PC1 was projected onto the initial structure for each system to graphically visualize the dominant motions of different regions in GPR97 during the simulations (Figure 4).

The principal dynamic motions of GPR97 mainly resided in its TM5, TM6, and TM7 regions. In the BCM–GPR97 system, TM6 and TM7 exhibited a weak outward motion and a weak inward motion, respectively. This is a feature of active GPR97 conformation [44]. However, TM5 showed an opposite motion trend compared to TM6, suggesting a low level of GPR97 activation. In the BCM–GPR97–G_o_ system, TM5 appeared to move outward and TM7 displayed an increased inward motion trend, indicating an elevated active state of GPR97. Furthermore, in the BCM–GPR97–palmitoylated G_o_ system, the intracellular half of TM5 and TM6 consistently oriented outward, and the inward motion of TM7 increased. These coupled motions thus locked the GPR97 in an active conformation. Furthermore, the overall movement tendency of GPR97 was minimal in the BCM–GPR97–palmitoylated G_o_ system, suggesting that GPR97 has the most stable conformation when in complex with the palmitoylated G_o_.

In the cortisol–GPR97 system, TM6 tended to move inward, indicating the low activity state of GPR97. In contrast, TM5 and TM6 exhibited outward motions while the upper half of TM7 started to move inward in the cortisol–GPR97–G_o_ system. In the cortisol–GPR97–palmitoylated G_o_ system, there was an increased outward motion of TM5 compared to that in the cortisol–GPR97–G_o_ system, suggesting enhanced GPR97 activity.

**Figure 5:**
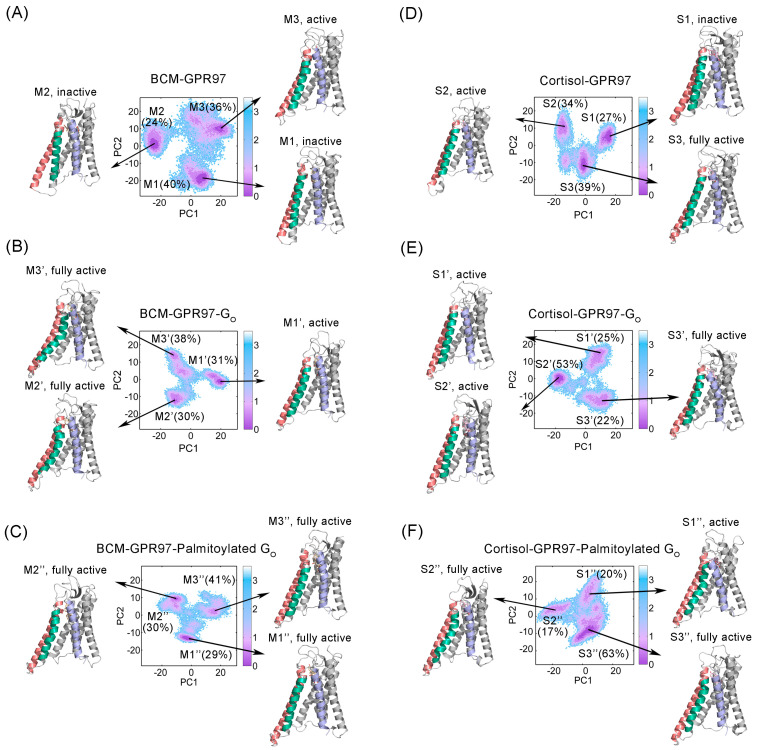
Projection of dominant metastates extracted from MSMs onto the GPR97 conformational landscape of BCM–GPR97 (**A**), BCM–GPR97–G_o_ (**B**), BCM–GPR97–palmitoylated G_o_ (**C**), cortisol–GPR97 (**D**), cortisol–GPR97–G_o_ (**E**), and cortisol–GPR97–palmitoylated G_o_ (**F**) systems generated using PCA. The representative GPR97 structure substates in BCM–GPR97 (M1, M2, and M3), BCM–GPR97–G_o_ (M1′, M2′, and M3′), BCM–GPR97–palmitoylated G_o_ (M1″, M2″, and M3″), cortisol–GPR97 (S1, S2, and S3), cortisol–GPR97–G_o_ (S1′, S2′, and S3′), and cortisol–GPR97–palmitoylated G_o_ (S1″, S2″, and S3″), along with their probabilities, are shown, with the TM5, TM6, and TM7 regions colored red, green, and purple, respectively.

To further explore the conformational dynamics of GPR97 during the simulations, we projected the overall conformation ensembles onto two-dimensional (2D) plots based on the two most dominant collective principal components (PC1 and PC2) of the PCA data. MSM analysis of the conformational landscape was performed using PyEMMA [45] to investigate the key conformational states of GPR97 (Figure 5). Our MSM models were confirmed to be Markovian using implied timescale tests (Figure S1) and the Chapman–Kolmogorov test (Figure S2).

Conformational ensembles of GPR97 were clustered into three MSM metastable states in each system, with the most distinguishing conformational differences of the TM5, TM6, and TM7 regions. The conformations of M1 and M2 in the BCM–GPR97 system exhibited the TM6 “in” conformation, implying their inactive states. M3, which accounted for 36% of the BCM–GPR97 conformational cluster, displayed a TM6 “out” active conformation. Notably, the conformational ensemble of GPR97 transformed to an active state in response to G_o_ binding. M2′ and M3′, accounting for 68%, reached their fully active states, as the intracellular end of TM6 shifted noticeably outward. In the BCM–GPR97–palmitoylated G_o_ system, the active conformations were further stabilized, shifting all three metastable substates (M1″, M2″, and M3″) towards their fully active forms.

In the cortisol–GPR97 system, the representative substate S1, which accounted for 27% of the GPR97 conformational cluster, presented an inactive structure. S2 (34%) and S3 (39%) exhibited active and a fully active states, respectively, which may have been related to the greater potency of cortisol (approximately threefold higher) than of BCM. In the BCM–GPR97–G_o_ system, S1′ and S2′, which accounted for 78% of the conformational clusters, exhibited active conformations, while S3 (22%) exhibited a fully active state. In the cortisol–GPR97–palmitoylated G_o_ system, all three substates (S1″, S2″, and S3″) displayed the active conformation, characterized by the outward movement of the intracellular end of TM5 and TM6, and an inward shift of TM7.

Collectively, the conformational landscape analyses using MSMs revealed that G_o_ binding stabilized the GPR97 active conformation, which could be enhanced by the palmitoylation of G_o_.

### 3.3. The Palmitoylation of G_o_ Strengthens the Interaction between GPR97 and Its Agonists

**Figure 6:**
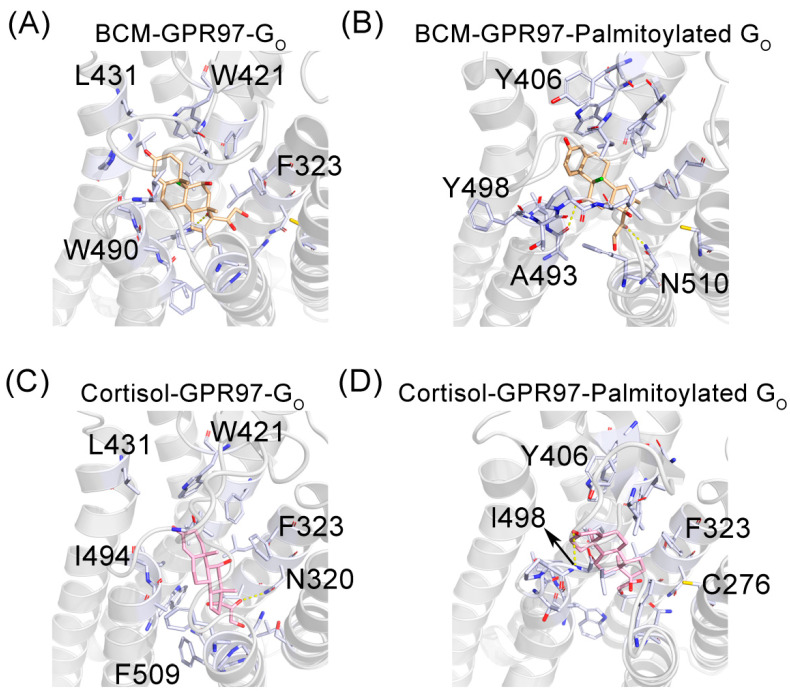
The detailed interactions of BCM (slate blue) and cortisol (pink) with GPR97 within BCM–GPR97–G_o_ (**A**), BCM–GPR97–palmitoylated G_o_ (**B**), cortisol–GPR97–G_o_ (**C**), and cortisol–GPR97–palmitoylated G_o_ (**D**) systems. The residues involved in the interactions with GPR97 are indicated by light-blue stick models. The hydrogen bonds are depicted as yellow lines.

To investigate the mechanism by which the palmitoylation of G_o_ stabilizes and enhances the active conformation of GPR97, we investigated the interaction between GPR97 and its agonists in the presence and absence of palmitoylation (Figure 6). In the BCM–GPR97–G_o_ system, there was one hydrogen bond and three weak hydrogen bonds between BCM and GPR97 (Figure 6A), while in the BCM–GPR97–palmitoylated G_o_ system, there were two hydrogen bonds and six weak hydrogen bonds between BCM and GPR97 (Figure 6B). Furthermore, in the BCM–GPR97–palmitoylated G_o_ system, BCM and GPR97 formed 39 hydrophobic contacts, as opposed to the 31 hydrophobic contacts in the BCM–GPR97–G_o_ system. Likewise, cortisol and GPR97 formed 1 hydrogen bond, 1 weak hydrogen bond, and 33 hydrophobic interactions in the cortisol–GPR97–G_o_ system (Figure 6C), but formed 1 hydrogen bond, 3 weak hydrogen bonds, and 45 hydrophobic contacts in the cortisol–GPR97–palmitoylated G_o_ system (Figure 6D). Taken together, the palmitoylation of G_o_ can promote the binding of agonist ligands to GPR97 and, thus, improve the activity of GPR97.

**Figure 7:**
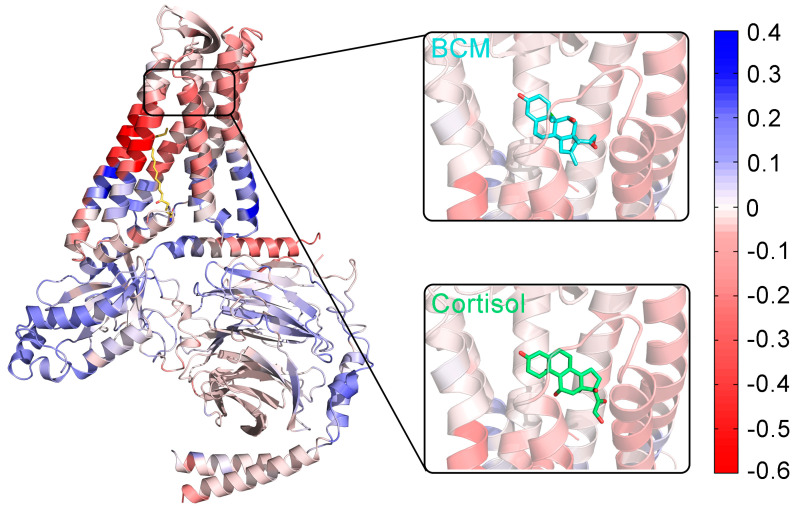
The allosteric effects of palmitoylation at the G_o_ C-tail on GPR97, calculated by AlloSigMA. GPR97 is colored according to per-residue allosteric free energy values (Δgi). The unit of free energy values is kcal/mol on the right-hand side of the color map.

As GPCRs are classic allosteric proteins [46], we further explored the coupling between the palmitoylated G_o_ and the BCM/cortisol ligand sites using energetic dynamics calculations. The allosteric free energy—i.e., the work exerted on residue i in the presence of the palmitoylation of G_o_—was analyzed using AlloSigMA [47,48,49,50,51] (Figure 7). GPR97 was colored based on the allosteric free energy values (Δgi). The color map on the right is taken to show the energy values: blue (positive Δgi) represents enhanced conformational changes, while red (negative Δgi) reflects suppressed conformational changes upon effector binding. The dynamics of white-colored residues were mostly unaffected by effector binding. It was observed that the allosteric free energy of residues in the ligand-binding pocket was negative. Such phenomena indicate that the palmitoylation chain has a stabilizing effect on residues around the ligands [52]. The stability of the ligand-binding pocket may contribute to the increased affinity of the ligands for GPR97.

### 3.4. Palmitoylation at the G_o_ C-Tail Reprograms the Structural Community and Allosteric Signal Network

**Figure 8:**
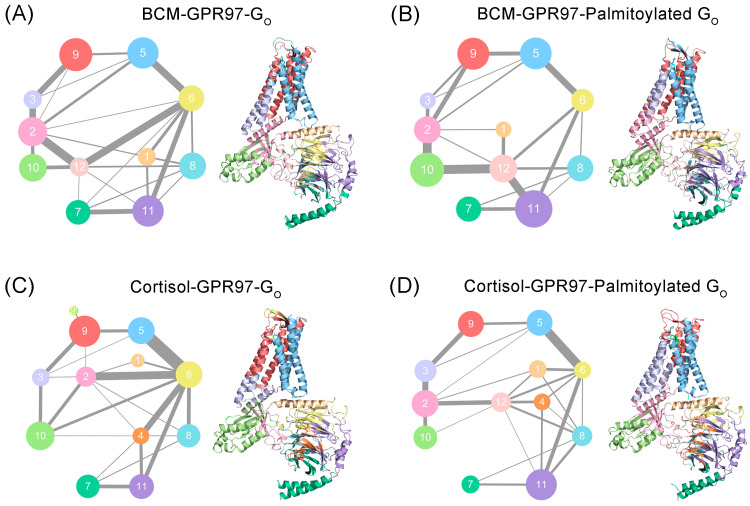
Colored community networks of BCM–GPR97–G_o_ (**A**), BCM–GPR97–palmitoylated G_o_ (**B**), cortisol–GPR97–G_o_ (**C**), and cortisol–GPR97–palmitoylated G_o_ (**D**). Each sphere represents an individual community with an area proportional to the number of residues it contains. The lines connecting different spheres visualize the intercommunity connections, while the thickness of the lines is proportional to the corresponding edge connectivity.

The propagation pathways of the allosteric signal within GPR97 were analyzed based on the Girvan–Newman algorithm. The variational coupling among the communities was quantitatively estimated. During the trajectory, residues within a cutoff distance of 4.5 Å for at least 75% of the simulation time were classified as part of the same communities, which were recognized as synergistic functional units within the overall protein. The visualized community network graphs provide clear depictions of the allosteric crosstalk paths and the corresponding intensities within GPR97 in different systems (Figure 8).

Distinct alterations in the topological characteristics and the intercommunity communications within the GPR97–G_o_ allosteric network were observed with and without the palmitoylation of G_o_. In the BCM–GPR97–G_o_ system, GPR97 mainly consists of Communities 3, 5, and 9. G_αo_ contains Communities 1, 2, 10, and 12 while G_βγ_ contains Communities 6, 7, 8, 11, and 12. Community 12, serving as the intersection of G_αo_ and G_βγ_, is involved in the composition of both G_αo_ and G_βγ_. In the BCM–GPR97–palmitoylated G_o_ system, the intracellular ends of TM5 and TM6 within GPR97 are incorporated into Community 2, and the linkage between Communities 2 and 9 is thickened relative to that in the BCM–GPR97–G_o_ system, indicating the strengthened interaction between G_αo_ and GPR97. For Community 12, the overall interaction with Communities 6, 8, and 11 of G_βγ_ is enhanced, suggesting an improved signal linkage between G_αo_ and G_βγ_ due to the palmitoylation.

Moreover, Community 9′, representing the extracellular loop (ECL), which is split out of Community 9 in the cortisol–GPR97–G_o_ system, is incorporated into Community 9 in the cortisol–GPR97–palmitoylated G_o_ system. This implies that the palmitoylation of G_o_ promotes the association of ECL with GPR97. In addition, in the cortisol–GPR97–G_o_ system, residues at the interface of G_αo_ and G_βγ_ are present in Community 2, whereas a new Community 12 is independent and responsible for the interaction between G_αo_ and G_βγ_ after the palmitoylation of Gα. Community 2 has a strong interaction with Community 6 and weak interaction with Communities 4 and 8 in the cortisol–GPR97–G_o_ system. For Community 12, the allosteric pathways with Communities 4 and 8 are enhanced, indicating that there exist extensive and balanced interactions between G_αo_ and G_βy_.

Collectively, the mechanism by which palmitoylation of G_o_ increases GPR97 activity lies in the enhancement of the internal stability of GPR97’s orthosteric site, as well as the promotion of signal flows within the G_o_ protein.

## 4. Discussion

GPCRs are the largest family of membrane proteins, and serve as leading targets of currently marketed drugs [49]. GPCRs comprise five main families in mammals [53]. The largest is the rhodopsin family, i.e., class A, with about 284 members in humans, followed by the aGPCR family, with 33 members, and then the glutamate family (class C), secretin family (class B), and frizzled family, with 22, 15, and 11 members, respectively [54]. GPR97 is a member of the aGPCR family, expressed in human granulocytes and endothelial cells of the vasculature. GPR97 triggers cyclic adenosine monophosphate (cAMP) by coupling with G_αo_, and actives the cAMP response element-binding protein (CREB), NF-κB, and small GTPases to modulate biological functions. A recent report has revealed that a palmitoylation presents at the C-tail end of G_o_ within the active GPR97–G_o_ complex, which has not yet been observed in other GPCRs [18]. The palmitoylation of the C-terminus of G_o_ contributes to its specific coupling to GPR97, leading to the high basal activity of GPR97. To elucidate the underlying mechanism, we carried out a comparative MD simulation study and computational analysis to obtain a dynamic conformational view [55,56,57,58].

RMSD and RMSF data revealed that the GPR97–palmitoylated G_0_ complex had more stable conformational dynamics than the unpalmitoylated G_0_ complex. DCCM analysis showed that the residue interactions within GPR97 in the GPR97–palmitoylated G_o_ system were the most stable, implying that the palmitoylation of G_o_ could limit the correlated motions between domains within GPR97. Based on PCA data, the conformational landscapes were analyzed using MSM. The dominant MSM metastable state indicated that GPR97 was preferred to be active when interacting with the palmitoylated G_o_. The interaction between ligands and GPR97 with and without palmitoylation of G_o_ was investigated, indicating that palmitoylation of G_o_ could improve the affinity of the ligands for GPR97. Furthermore, we found that the palmitoylation of G_o_ allosterically strengthened internal interactions with G_o_, enhanced the coupling between G_o_ and GPR97, and stabilized the ligand-binding cavity within GPR97. The loss-of-function mutation of residue 503 in GPR56—which belongs to the same aGPCR family as GPR97—was detected in bilateral frontoparietal polymicrogyria (BFPP) [59,60,61]. Given that G_o_ palmitoylation can stabilize the active conformation of GPR97, we speculate that this mutation may disrupt the interaction of G_o_ palmitoylation with GPCR, as the residue is located around the G_o_ palmitoylation site.

Taken together, our findings provide the first dynamic conformational insights into the palmitoylation of G_o_ interacting with GPR97—a poorly characterized aGPCR. In future studies, this may open new possibilities for exploring the regulation of GPCRs’ functions through PTMs of G proteins.

## Data Availability

Not applicable.

## Supplementary Materials

The following supporting information can be downloaded at: https://www.mdpi.com/article/10.3390/pharmaceutics14091856/s1, Figure S1: The result of implied timescale test for MSMs in BCM–GPR97 (A), BCM–GPR97–GO (B), BCM–GPR97–Palmitoylated GO (C), Cortisol–GPR97 (D), Cortisol–GPR97–GO (E), and Cortisol–GPR97–Palmitoylated GO (F) systems; Figure S2: The results of the Chapman-Kolmogorov test of metastable states for BCM-GPR97 (A), BCM–GPR97–GO (B), BCM–GPR97–Palmitoylated GO (C), Cortisol–GPR97 (D), Cortisol–GPR97–GO (E), and Cortisol–GPR97–Palmitoylated GO (F) systems system.

## References

[1] Gupte J., Swaminath G., Danao J., Tian H., Li Y., Wu X. (2012). Signaling property study of adhesion G-protein-coupled receptors. FEBS Lett..

[2] Wang Y., Li M., Liang W., Shi X., Fan J., Kong R., Liu Y., Zhang J., Chen T., Lu S. (2022). Delineating the activation mechanism and conformational landscape of a class B G protein-coupled receptor glucagon receptor. Comput. Struct. Biotechnol. J..

[3] Monk K.R., Naylor S.G., Glenn T.D., Mercurio S., Perlin J.R., Dominguez C., Moens C.B., Talbot W.S. (2009). A G protein-coupled receptor is essential for Schwann cells to initiate myelination. Science.

[4] Stoveken H.M., Hajduczok A.G., Xu L., Tall G.G. (2015). Adhesion G protein-coupled receptors are activated by exposure of a cryptic tethered agonist. Proc. Natl. Acad. Sci. USA.

[5] Araç D., Boucard A.A., Bolliger M.F., Nguyen J., Soltis S.M., Südhof T.C., Brunger A.T. (2012). A novel evolutionarily conserved domain of cell-adhesion GPCRs mediates autoproteolysis. EMBO J..

[6] Weis W.I., Kobilka B.K. (2018). The Molecular Basis of G Protein-Coupled Receptor Activation. Annu. Rev. Biochem..

[7] Giulio M., Silvia A.-G., Timothy C., Luigi G.F. (2020). A combined activation mechanism for the glucagon receptor. Proc. Natl. Acad. Sci. USA.

[8] Purcell R.H., Hall R.A. (2018). Adhesion G Protein-Coupled Receptors as Drug Targets. Annu. Rev. Pharmacol. Toxicol..

[9] Wang J., Miao Y., Wicklein R., Sun Z., Wang J., Jude K.M., Fernandes R.A., Merrill S.A., Wernig M., Garcia K.C. (2022). RTN4/NoGo-receptor binding to BAI adhesion-GPCRs regulates neuronal development. Cell.

[10] Bassilana F., Nash M., Ludwig M.-G. (2019). Adhesion G protein-coupled receptors: Opportunities for drug discovery. Nat. Rev. Drug Discov..

[11] Folts C.J., Giera S., Li T., Piao X. (2019). Adhesion G Protein-Coupled Receptors as Drug Targets for Neurological Diseases. Trends Pharmacol. Sci..

[12] Guihurt Santiago J., Herrera Camacho R., Flores Otero J. (2016). Determining the Role of Adhesion G-Protein Coupled Receptors in Retinoblastoma. Investig. Ophthalmol. Vis. Sci..

[13] Paavola K.J., Hall R.A. (2012). Adhesion G Protein-Coupled Receptors: Signaling, Pharmacology, and Mechanisms of Activation. Mol. Pharmacol..

[14] Fang W., Wang Z., Li Q., Wang X., Zhang Y., Sun Y., Tang W., Ma C., Sun J., Li N. (2018). Gpr97 Exacerbates AKI by Mediating Sema3A Signaling. J. Am. Soc. Nephrol..

[15] Wang J.-J., Zhang L.-L., Zhang H., Shen C.-L., Lu S., Kuang Y., Wan Y., Wang W., Yan H., Dang S. (2013). Gpr97 is essential for the follicular versus marginal zone B-lymphocyte fate decision. Cell Death Dis..

[16] Hsiao C.-C., Chu T.-Y., Wu C.-J., van den Biggelaar M., Pabst C., Hébert J., Kuijpers T.W., Scicluna B.P., I K.-Y., Chen T.-C. (2018). The Adhesion G Protein-Coupled Receptor GPR97/ADGRG3 Is Expressed in Human Granulocytes and Triggers Antimicrobial Effector Functions. Front. Immunol..

[17] Tobias L., Gabriela A., Jörg H. (2013). Sticky Signaling—Adhesion Class G Protein—Coupled Receptors Take the Stage. Sci. Signal..

[18] Ping Y.-Q., Mao C., Xiao P., Zhao R.-J., Jiang Y., Yang Z., An W.-T., Shen D.-D., Yang F., Zhang H. (2021). Structures of the glucocorticoid-bound adhesion receptor GPR97–Go complex. Nature.

[19] Linder M.E., Deschenes R.J. (2007). Palmitoylation: Policing protein stability and traffic. Nat. Rev. Mol. Cell Biol..

[20] Morello J.-P., Bouvier M. (1996). Palmitoylation: A post-translational modification that regulates signalling from G-protein coupled receptors. Biochem. Cell Biol..

[21] Qanbar R., Bouvier M. (2003). Role of palmitoylation/depalmitoylation reactions in G-protein-coupled receptor function. Pharmacol. Ther..

[22] Chen C.A., Manning D.R. (2001). Regulation of G proteins by covalent modification. Oncogene.

[23] Sikarwar A.S., Bhagirath A.Y., Dakshinamurti S. (2019). Effects of Post-translational Modifications on Membrane Localization and Signaling of Prostanoid GPCR–G Protein Complexes and the Role of Hypoxia. J. Membr. Biol..

[24] Lu S., Zhang J. (2019). Small Molecule Allosteric Modulators of G-Protein-Coupled Receptors: Drug-Target Interactions. J. Med. Chem..

[25] Wang Y., Yu Z., Xiao W., Lu S., Zhang J. (2021). Allosteric binding sites at the receptor—Lipid bilayer interface: Novel targets for GPCR drug discovery. Drug Discov. Today.

[26] Lomize M.A., Pogozheva I.D., Joo H., Mosberg H.I., Lomize A.L. (2012). OPM database and PPM web server: Resources for positioning of proteins in membranes. Nucleic Acids Res..

[27] Lee J., Cheng X., Swails J.M., Yeom M.S., Eastman P.K., Lemkul J.A., Wei S., Buckner J., Jeong J.C., Qi Y. (2016). CHARMM-GUI Input Generator for NAMD, GROMACS, AMBER, OpenMM, and CHARMM/OpenMM Simulations Using the CHARMM36 Additive Force Field. J. Chem. Theory Comput..

[28] Maier J.A., Martinez C., Kasavajhala K., Wickstrom L., Hauser K.E., Simmerling C. (2015). ff14SB: Improving the Accuracy of Protein Side Chain and Backbone Parameters from ff99SB. J. Chem. Theory Comput..

[29] Wang J., Wolf R.M., Caldwell J.W., Kollman P.A., Case D.A. (2004). Development and testing of a general amber force field. J. Comput. Chem..

[30] Jorgensen W.L., Chandrasekhar J., Madura J.D., Impey R.W., Klein M.L. (1983). Comparison of simple potential functions for simulating liquid water. J. Chem. Phys..

[31] Darden T., York D., Pedersen L. (1993). Particle mesh Ewald: An N log(N) method for Ewald sums in large systems. J. Chem. Phys..

[32] Ryckaert J.-P., Ciccotti G., Berendsen H.J.C. (1977). Numerical integration of the cartesian equations of motion of a system with constraints: Molecular dynamics of n-alkanes. J. Comput. Phys..

[33] Hünenberger P.H., Mark A.E., van Gunsteren W.F. (1995). Fluctuation and Cross-correlation Analysis of Protein Motions Observed in Nanosecond Molecular Dynamics Simulations. J. Mol. Biol..

[34] Amadei A., Linssen A.B.M., Berendsen H.J.C. (1993). Essential dynamics of proteins. Proteins Struct. Funct. Bioinform..

[35] Scherer M.K., Trendelkamp-Schroer B., Paul F., Pérez-Hernández G., Hoffmann M., Plattner N., Wehmeyer C., Prinz J.-H., Noé F. (2015). PyEMMA 2: A Software Package for Estimation, Validation, and Analysis of Markov Models. J. Chem. Theory Comput..

[36] Bowman G.R., Ensign D.L., Pande V.S. (2010). Enhanced modeling via network theory: Adaptive sampling of Markov state models. J. Chem. Theory Comput..

[37] Prinz J.-H., Wu H., Sarich M., Keller B., Senne M., Held M., Chodera J.D., Schütte C., Noé F. (2011). Markov models of molecular kinetics: Generation and validation. J. Chem. Phys..

[38] Chodera J.D., Singhal N., Pande V.S., Dill K.A., Swope W.C. (2007). Automatic discovery of metastable states for the construction of Markov models of macromolecular conformational dynamics. J. Chem. Phys..

[39] Bowman G.R., Huang X., Pande V.S. (2009). Using generalized ensemble simulations and Markov state models to identify conformational states. Methods.

[40] Eargle J., Luthey-Schulten Z. (2012). NetworkView: 3D display and analysis of protein RNA interaction networks. Bioinformatics.

[41] Ni D., Wei J., He X., Rehman A.U., Li X., Qiu Y., Pu J., Lu S., Zhang J. (2021). Discovery of cryptic allosteric sites using reversed allosteric communication by a combined computational and experimental strategy. Chem. Sci..

[42] Sethi A., Eargle J., Black A.A., Luthey-Schulten Z. (2009). Dynamical networks in tRNA:protein complexes. Proc. Natl. Acad. Sci. USA.

[43] Dalton J.A.R., Lans I., Giraldo J. (2015). Quantifying conformational changes in GPCRs: Glimpse of a common functional mechanism. BMC Bioinform..

[44] Mansoor S., Kayık G., Durdagi S., Sensoy O. (2022). Mechanistic insight into the impact of a bivalent ligand on the structure and dynamics of a GPCR oligomer. Comput. Struct. Biotechnol. J..

[45] Lu S., He X., Yang Z., Chai Z., Zhou S., Wang J., Rehman A.U., Ni D., Pu J., Sun J. (2021). Activation pathway of a G protein-coupled receptor uncovers conformational intermediates as targets for allosteric drug design. Nat. Commun..

[46] Mirka V.M., Ramachandran R., Laprairie R. (2022). Allosteric modulation of tethered ligand-activated G protein-coupled receptors. Allosteric Modulation of G Protein-Coupled Receptors.

[47] Guarnera E., Tan Z.W., Zheng Z., Berezovsky I.N. (2017). AlloSigMA: Allosteric signaling and mutation analysis server. Bioinformatics.

[48] Wall M.E. (2006). Ligand Binding, Protein Fluctuations, and Allosteric Free Energy. AIP Conf. Proc..

[49] Guarnera E., Berezovsky I.N. (2019). Toward Comprehensive Allosteric Control over Protein Activity. Structure.

[50] Guarnera E., Berezovsky I.N. (2019). On the perturbation nature of allostery: Sites, mutations, and signal modulation. Curr. Opin. Struct. Biol..

[51] Guarnera E., Berezovsky I.N. (2016). Structure-Based Statistical Mechanical Model Accounts for the Causality and Energetics of Allosteric Communication. PLoS Comput. Biol..

[52] Tan Z.W., Guarnera E., Tee W.-V., Berezovsky I.N. (2020). AlloSigMA 2: Paving the way to designing allosteric effectors and to exploring allosteric effects of mutations. Nucleic Acids Res..

[53] Hauser A.S., Attwood M.M., Rask-Andersen M., Schiöth H.B., Gloriam D.E. (2017). Trends in GPCR drug discovery: New agents, targets and indications. Nat. Rev. Drug Discov..

[54] Cong X., Zhang X., Liang X., He X., Tang Y., Zheng X., Lu S., Zhang J., Chen T. (2022). Delineating the conformational landscape and intrinsic properties of the angiotensin II type 2 receptor using a computational study. Comput. Struct. Biotechnol. J..

[55] Lu S., Ni D., Wang C., He X., Lin H., Wang Z., Zhang J. (2019). Deactivation Pathway of Ras GTPase Underlies Conformational Substates as Targets for Drug Design. ACS Catal..

[56] Lu S., Chen Y., Wei J., Zhao M., Ni D., He X., Zhang J. (2021). Mechanism of allosteric activation of SIRT6 revealed by the action of rationally designed activators. Acta Pharm. Sin. B.

[57] Qiu Y., Yin X., Li X., Wang Y., Fu Q., Huang R., Lu S. (2021). Untangling Dual-Targeting Therapeutic Mechanism of Epidermal Growth Factor Receptor (EGFR) Based on Reversed Allosteric Communication. Pharmaceutics.

[58] Li X., Wang C., Peng T., Chai Z., Ni D., Liu Y., Zhang J., Chen T., Lu S. (2021). Atomic-scale insights into allosteric inhibition and evolutional rescue mechanism of Streptococcus thermophilus Cas9 by the anti-CRISPR protein AcrIIA6. Comput. Struct. Biotechnol. J..

[59] Piao X., Hill R.S., Bodell A., Chang B.S., Basel-Vanagaite L., Straussberg R., Dobyns W.B., Qasrawi B., Winter R.M., Innes A.M. (2004). G Protein-Coupled Receptor-Dependent Development of Human Frontal Cortex. Science.

[60] Piao X., Chang B.S., Bodell A., Woods K., BenZeev B., Topcu M., Guerrini R., Goldberg-Stern H., Sztriha L., Dobyns W.B. (2005). Genotype–Phenotype analysis of human frontoparietal polymicrogyria syndromes. Ann. Neurol..

[61] Chiang N.-Y., Hsiao C.-C., Huang Y.-S., Chen H.-Y., Hsieh I.-J., Chang G.-W., Lin H.-H. (2011). Disease-associated GPR56 mutations cause bilateral frontoparietal polymicrogyria via multiple mechanisms. J. Biol. Chem..

