# Supplementary material for: Mechanistic Understanding of the Palmitoylation of Go Protein in the Allosteric Regulation of Adhesion Receptor GPR97
Source: Pharmaceutics. 2022 Sep 2;14(9):1856. doi: 10.3390/pharmaceutics14091856 (PMC9504338; doi:10.3390/pharmaceutics14091856)
Supplement: Supplementary file 1 [file pharmaceutics-14-01856-s001.zip › pharmaceutics-1847310-supplementary.pdf]

Supplemental Information

Mechanistic Understanding of the Palmitoylation of the G_o Protein in the Allosteric Regulation of Adhesion Receptor GPR97

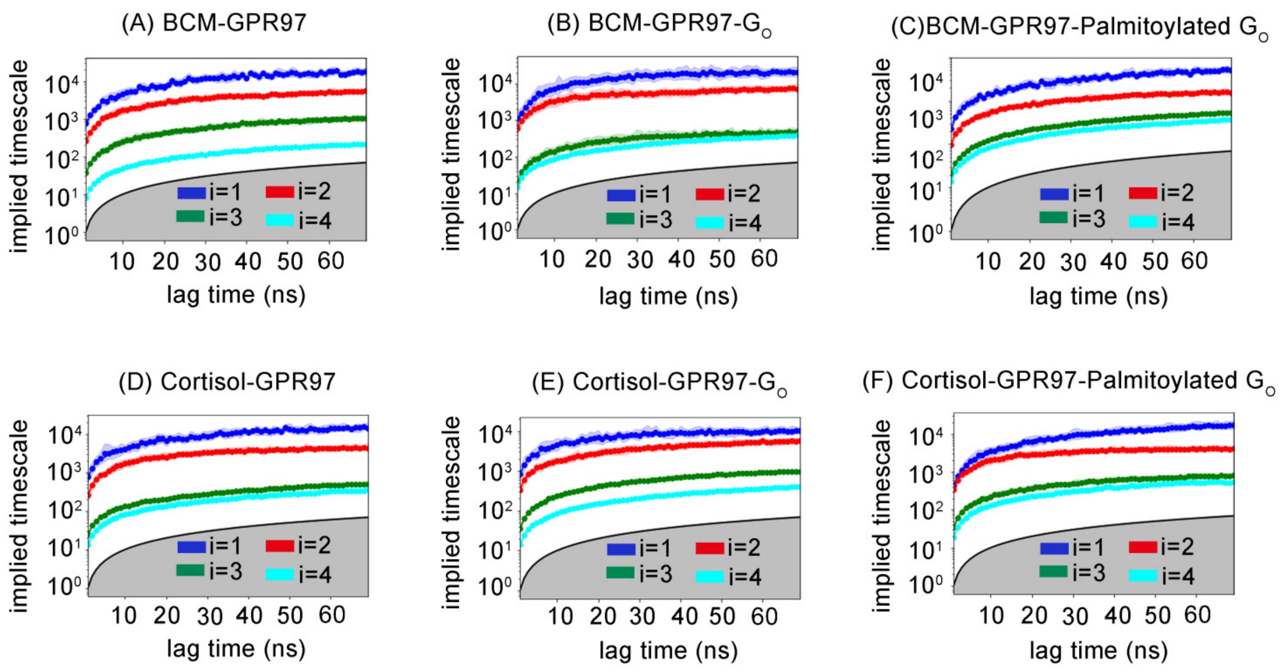

Figure S1. The result of implied timescale test for MSMs in BCM–GPR97 **(A)**, BCM–GPR97–G_o **(B)**, BCM–GPR97–Palmitoylated G_o **(C),** Cortisol–GPR97 **(D)**, Cortisol–GPR97–G_o **(E)**, and Cortisol–GPR97–Palmitoylated G_o **(F)** systems. Blue, red, green, and cyan lines showed the timescale τ_1, τ_2, τ_3 and τ_4 as a function of lag times. Black line represents x=y in logarithmic coordinates.

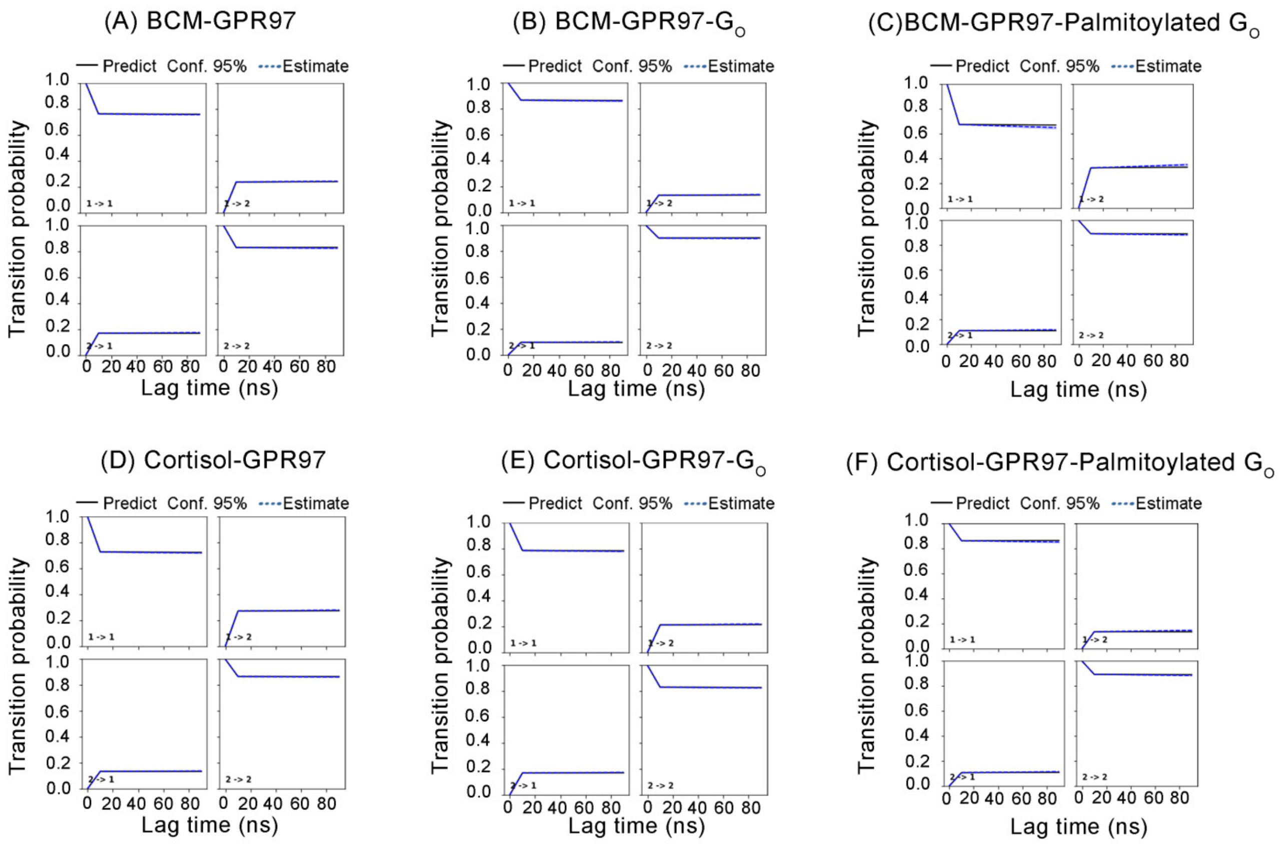

Figure S2. The results of the Chapman-Kolmogorov test of metastable states for BCM-GPR97 **(A)**, BCM–GPR97–Go **(B)**, BCM–GPR97–Palmitoylated Go **(C),** Cortisol–GPR97 **(D)**, Cortisol–GPR97–Go **(E)**, and Cortisol–GPR97–Palmitoylated Go **(F)** systems system. Shown are data for MSM (black line) and the observed trajectory (blue dotted line, with estimated error).
